# Pseudoaneurysm of a high-division anterior tibial artery following primary TKA

**DOI:** 10.1007/s00132-016-3373-3

**Published:** 2017-01-03

**Authors:** Ran Zhao, Yang Li, Yanqing Liu, Ke Zhang, Zhongjun Liu

**Affiliations:** 1 Peking University Third Hospital, Beijing, China; 2 Department of Orthopaedic Surgery, Peking University Third Hospital, 49 North Gardon Road, Haidian District, Beijing, China

**Keywords:** Hematoma, Anterior tibial artery, Risk factors, Thrombosis, Embolism, Hämatom, A. tibialis anterior, Risikofaktoren, Thrombose, Embolie

## Abstract

Arterial complications following total knee arthroplasty are rare but carry a high risk of significant morbidity and mortality. Herein, the authors report a case of pseudoaneurysm in a branch of a high-division anterior tibial artery caused by laceration by the retractor following primary total knee arthroplasty in a 72-year-old woman. The patient presented with an unexplained hematoma and an enlarging swelling, with pallor and acute hemorrhage on the first postoperative day. The diagnosis was confirmed by arteriography and the patient was successfully treated using endovascular embolization without long-term complications. Because of the high risk of progression to a potentially limb- or life-threatening condition, it is important to discuss the risk factors for this complication, as well as its early diagnosis and treatment methods.

**Electronic supplementary material:**

The online version of this article (doi: 10.1007/s00132-016-3373-3) contains two videos on diagnosis and treatment of the pseudoaneurysm. The article and the supplementary material are available in the electronic archive at http://www.springermedizin.de/der-orthopaede.

## Zusammenfassung

Arterielle Komplikationen nach totaler Kniearthroplastik sind selten, weisen aber eine hohes Risiko erheblicher Morbidität und Mortalität auf. In der vorliegenden Arbeit berichten die Autoren über den Fall eines Pseudoaneurysmas, das einen hoch abgehenden Ast der A. tibialis anterior betraf und durch einen vom Retraktor verursachten Geweberiss nach primärer totaler Kniearthroplastik bei einer 72-jährigen Patientin entstand. Die Patientin wies am ersten postoperativen Tag ein Hämatom unklarer Genese und eine zunehmende Schwellung mit Blässe und akuter Hämorrhagie auf. Mittels Arteriographie wurde die Diagnose bestätigt und die Patientin erfolgreich durch eine endovaskuläre Embolisation ohne Langzeitkomplikationen behandelt. Wegen des hohen Risikos der Progression zu einer potenziell für die Extremität oder das Leben bedrohlichen Situation ist es wichtig, die Risikofaktoren dieser Komplikation zu erörtern sowie Verfahren zur frühzeitigen Diagnosestellung und Behandlung darzustellen.

Arterial complications associated with total knee arthroplasty (TKA) are rare, with a reported incidence of only 0.06 to 0.20% [1]. Such arterial complications include thrombosis, embolism, pseudoaneurysm, arteriovenous fistula, and arterial transection [2, 3]. These complications may occur intraoperatively, immediately postoperatively, or in the late postoperative period. The clinical symptoms are nonspecific, but once an arterial complication has been diagnosed, it must be treated as soon as possible to prevent potentially devastating sequelae.

Most such vascular injuries involve the popliteal artery, because of its proximity to the surgical site during TKA. The anterior tibial artery (ATA) is a terminal branch of the popliteal artery and usually divides below the articular surface of the tibial plateau. The presence of a high-division ATA may increase the risk of intraoperative vascular injury [4]. Herein, the authors report the case of a pseudoaneurysm arising from a high-division ATA during primary TKA that was successfully treated with endovascular embolization without long-term complications.

## Case report

**Fig. 1:**
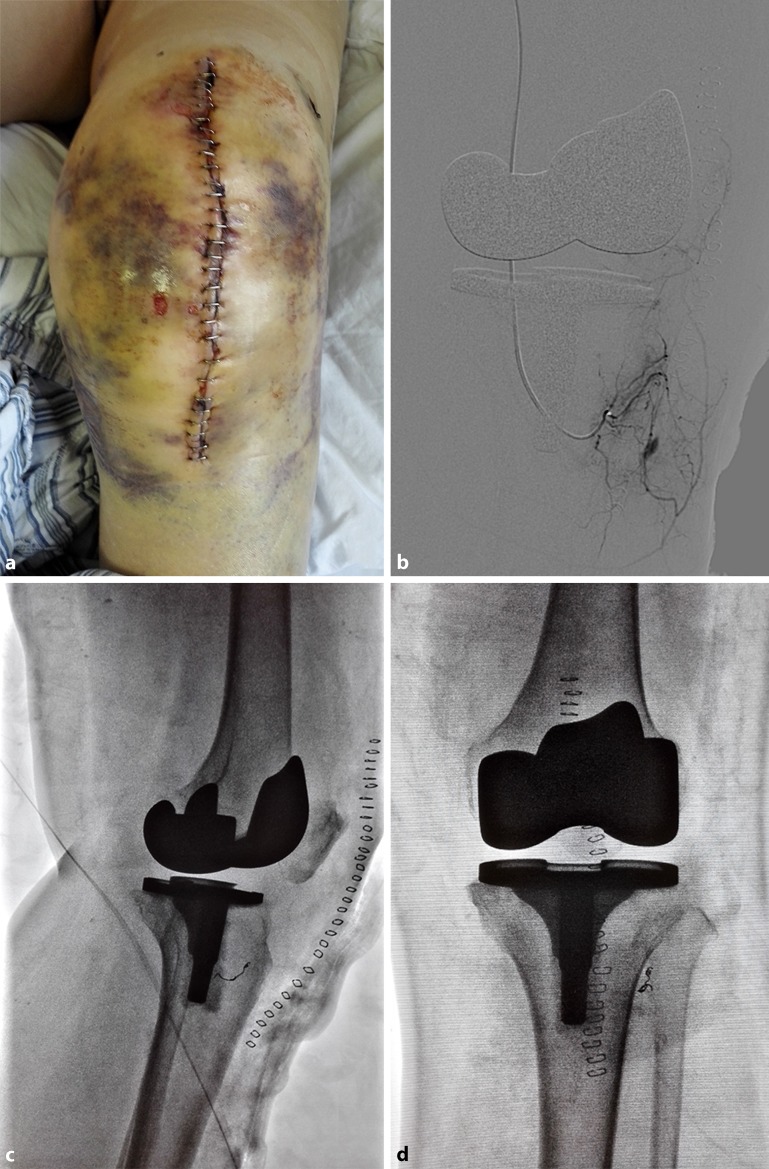
**a** An unexplained hematoma and an enlarging swelling developed on the left knee on the first postoperative day. **b** Urgent angiography showed a pseudoaneurysm arising from a branch of the anterior tibial artery, which is a high division of the popliteal artery above the knee joint. **c,** **d** The pseudoaneurysm was treated with microcoil embolization

A 72-year-old woman developed severe left knee joint osteoarthritis with 20° flexion deformity. The patient’s Hospital for Special Surgery (HSS) score was 43 and her Western Ontario and McMaster Universities (WOMAC) score was 48. The patient underwent left primary TKA using a posterior-stabilized prosthesis (Genesis II™; Smith & Nephew, Memphis, TN, USA) with application of a tourniquet, which was not released until after wound closure. A standard medial parapatellar approach was used under intraspinal anesthesia. The surgery proceeded without any apparent intraoperative complications. Immediately postoperatively, the patient received thromboprophylaxis in the form of 100 mg of oral aspirin 12 h after surgery, local application of a compression bandage, and ankle pump exercises as physical therapy. During the night after the surgery, the wound bled slightly (drainage of 100 ml). The patient’s hemoglobin concentration was 89 g/L on the first postoperative morning (20 h after surgery) compared with 116 g/L preoperatively, but she had no complaints except pain (visual analog scale score of 3). At 24 h postoperatively, the compression bandage and drainage tube were removed. At 30 h postoperatively, the patient developed unexplained serious incisional errhysis, a hematoma, and an enlarging swelling on the left knee (Fig. 1a). The patient also exhibited pallor and palpitation, but all distal pulses remained palpable. The hemoglobin concentration at this time was 51 g/L, indicating acute hemorrhage. Urgent angiography under local anesthesia showed a pseudoaneurysm arising from a branch of the ATA, which is a high division of the popliteal artery above the knee joint (Fig. 1b; Video 1, Electronic Supplementary Material). The pseudoaneurysm was treated with endovascular embolization 31 h postoperatively. The feeding vessel was selectively catheterized and embolization was completed by covering the neck of the pseudoaneurysm with a 4 × 2 mm Embolization Microcoil^TM^ (COOK, Bloomington, IN, USA). Screening angiographs confirmed that the pseudoaneurysm was successfully sealed off (Fig. 1c, d; Video 2, Electronic Supplementary Material) and the incisional errhysis immediately stopped. The anemia was resolved by transfusion of 4 IU of suspended red blood cells and the hemoglobin concentration increased to 78 g/L at 45 h after surgery. Routine blood tests were repeated each day and the hemoglobin concentration remained at >80 g/L. The patient was discharged 1 week after surgery, at which time the hematoma and swelling had resolved and the hemoglobin concentration was 85 g/L. At the final follow-up 12 months postoperatively, the patient had experienced no recurrence of symptoms or neurologic deficits, and there was no necrosis around the embolism site; the patient could walk and perform full extension with 110° flexion, had a HSS score of 84, and a WOMAC score of 12.

## Discussion

A pseudoaneurysm develops secondary to rupture of the vascular wall, producing a pulsatile mass confined by a fibrous capsule. Without timely diagnosis and treatment, the pseudoaneurysm can repeatedly rupture and bleed, leading to serious complications in some cases [5]. Development of a pseudoaneurysm during TKA is rare but can significantly lengthen the hospital stay and increase hospital costs. It may also lead to serious complications such as knee stiffness, amputation, or death [6]. Few reports to date have described pseudoaneurysms arising from the ATA in association with TKA, perhaps because the ATA usually arises from the popliteal artery lower than the level of the tibial bone cut. Gupta et al. [7] reported a case of a pseudoaneurysm involving a branch of the ATA after TKA; the pseudoaneurysm was successfully treated by percutaneous embolization with a microcoil. Verma et al. [2] reported a case of a pseudoaneurysm of a branch of the ATA following TKA, and this pseudoaneurysm was also treated with coil embolization. The present report is the first to describe a pseudoaneurysm in a branch of a high-division ATA following primary TKA. In this case, the branch position of the high-division ATA may have been high enough to readily cause a direct injury.

Previous studies have determined the prevalence and prognosis of many types of vascular injuries during TKA based on large samples. Calligaro et al. [8] investigated a sample of 13,618 TKA procedures (including 1665 revisions) and found 24 arterial complications (0.17%); 3 were popliteal artery transections, 5 were popliteal artery pseudoaneurysms, and 16 were solely ischemic complications. Padegimas et al. [1] reported 13 vascular complications among 9951 TKAs (0.13%); in their study, all 13 patients with vascular complications had undergone vascular interventions: 9 stent placements, 2 endarterectomies, 1 thrombectomy, and 1 bypass. One patient sustained a peroneal nerve injury, and three developed persistent stiffness postoperatively that improved after manipulation.

**Fig. 2:**
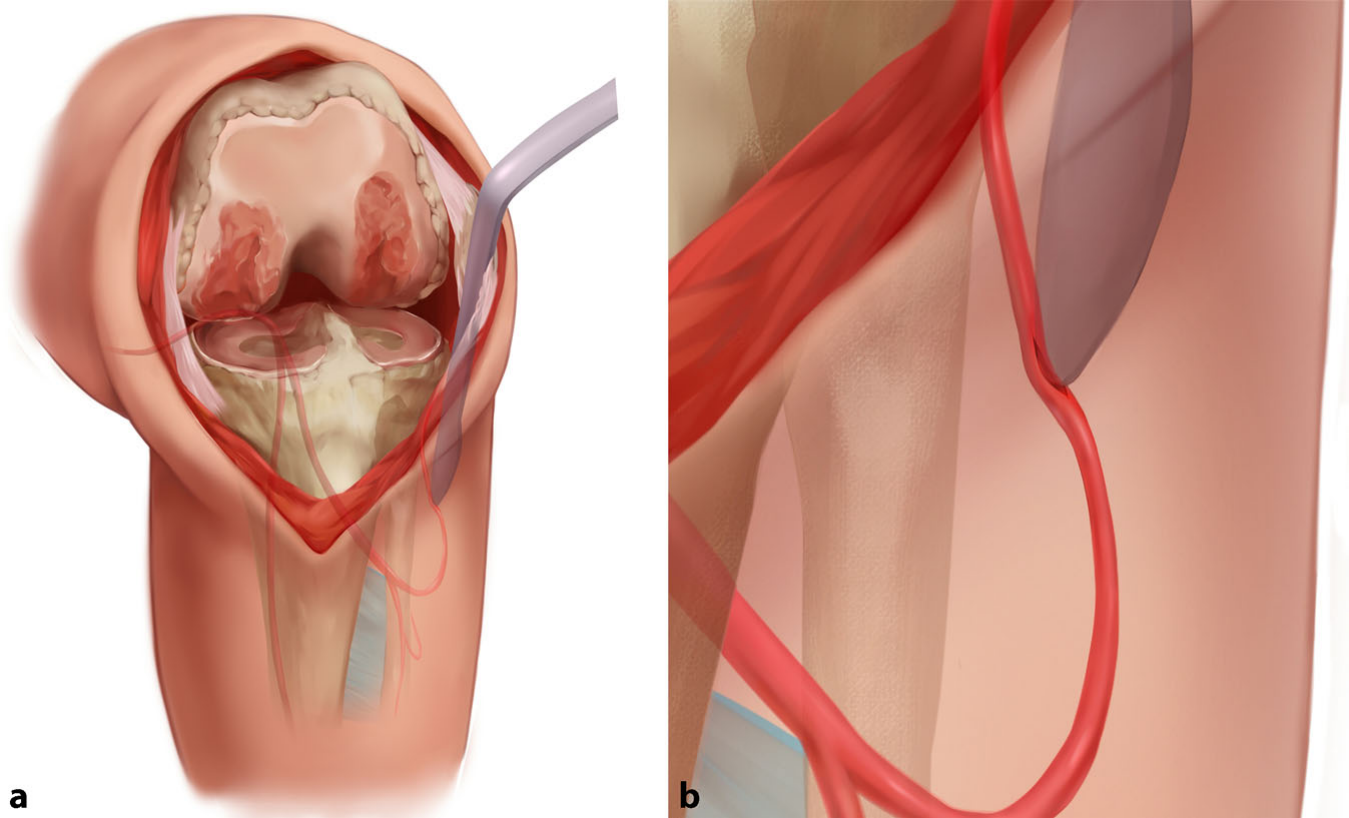
**a** A suspected high-division anterior tibial artery (ATA) moved up the branch vessel position. The mechanism of vascular injury was laceration of a branch of the high-division ATA along the subperiosteum by the point of the retractor during retraction of the lateral soft tissue, as shown in (**b**). This may have occurred if the retractor was inserted too deeply or at an incorrect angle

Herein, the authors have reported a pseudoaneurysm in a branch of a high-division ATA and consider that the vessel was lacerated by the retractor during retraction of the patella (Fig. 2). The surgery was successfully performed and the injury was not within the scope of the surgical field; it occurred lower than the level of the tibial bone cut. With respect to the cause of the pseudoaneurysm, the authors did not release the subperiosteum until reaching the injury plane during exposure of the lateral tibia, and only placed the retractor in the soft tissue to expose the surgical field. The authors believe that the laceration occurred during retraction of the lateral soft tissue, which may have occurred if the retractor was inserted too deeply or at an incorrect angle. Pseudoaneurysms usually develop after arterial laceration or transection caused by direct injury by sharp instruments [9, 10]. With the exception of a misplaced retractor, any instrument, including a vibrating saw, scalpel blade, or diathermy device, could lead to direct injury. The damage often occurs during resection of the posterior femoral condyles or proximal tibia, or during release of the posterior capsule [11]. If the posterior retractor is inserted lateral to the midline or >1 cm into the soft tissue, the risk of vascular injury increases [11]. Anatomic abnormalities of the ATA can also increase the risk of intraoperative injury. The ATA is identified as the first branch that arises at or above the articular surface of the tibial plateau; another landmark is the presence of the bifurcation proximal to the lower border of the popliteus muscle [12]. In a study by Klecker et al. [4], the incidence of a high-division ATA was 2.1%. In such cases, the risk of vascular trauma may increase during proximal tibial resection in TKA.

In addition to direct injury to a blood vessel, application of a tourniquet is another mechanism of arterial complications. The tourniquet can reduce intraoperative bleeding and facilitate a dry bone surface for the placement of cemented components. However, many studies have shown that tourniquet application contributes to the development of arterial complications in TKA, usually at the level of the superficial femoral artery [8, 10, 13]. Fixation of the superficial femoral artery by a tourniquet can lead to tears in the arterial intimal wall with subsequent formation of an occlusive thrombus. If the patient has preexisting vascular disease, the tourniquet could lead to fracture of an atheromatous plaque and subsequent arterial embolism. Considering that tourniquets are not essential in TKA, especially for high-risk patients, the need for their use should be evaluated in the preoperative phase [3]. Arterial complications caused by tourniquets usually occur at the level of the superficial femoral artery [3]; no reports have described involvement of the ATA.

Extreme joint positioning, release of severe flexion contractures and subsequent traction, and thermal injury from cementing have been described as other mechanisms of arterial complications [1, 14]. The formation of an extraosseous cement granuloma produced by wear products of acrylic cement could lead to late postoperative development of a pseudoaneurysm [15]. Patients with a history of peripheral arterial disease may also have a high risk of arterial complications [3]. The risk of arterial injury after revision TKA is twice that after a primary procedure because blood vessels may be encased by fibrous scar tissue, rendering them more vulnerable to indirect or direct injury [8, 16]. Other risk factors include weight loss, renal failure, coagulopathy, and metastatic cancer [6].

The symptoms of a pseudoaneurysm are very nonspecific and include unusual pain, unexplained hematoma formation and swelling of the knee, acute hemorrhage, and limited motion of the knee. The lower limb may exhibit pallor and paresthesia; these symptoms, together with decreased or absent pulses, strongly suggest vascular injury [16]. The presence of distal pulses may seem to make the diagnosis of arterial injury less probable. Color Doppler ultrasound is an initial imaging technique with which to diagnose vascular injury. When a noninvasive test cannot clarify the diagnosis, however, angiography may be required [16]. Angiography can show the exact location of the pseudoaneurysm and vascular malformation, and it can be used for therapeutic purposes by stopping the bleeding once discovered [17]. Computed tomography and magnetic resonance angiography are not usually considered for diagnosis of artifacts caused by the prostheses, while magnetic resonance imaging can be used to identify an aberrant ATA preoperatively [4].

Treatment of a pseudoaneurysm includes ultrasound-guided compression repair, percutaneous embolization, endovascular stenting, or open surgical repair. Ultrasound-guided compression repair has been largely superseded by other techniques. Percutaneous embolization under local anesthesia (comprising thrombin injection or microcoils) has a low rate of complications and a favorable prognosis [5]. In a study by Kang et al. [18], 82 of 83 pseudoaneurysms were successfully treated by percutaneous ultrasound-guided thrombin injection. A limitation of thrombin injection is that if the thrombin escapes to the native arterial circulation, vascular thrombosis or emboli can occur [5]. Many studies have shown that high-selectivity microcoil embolization under angiography can successfully seal the pseudoaneurysm without long-term complications [2, 19, 20]. In the present case, microcoil embolization rather than stenting was used for the pseudoaneurysm, which was situated at the branch of the ATA. It would have been difficult to use a stent, and a stent placed in the popliteal fossa could fracture or migrate when the knee joint is in full flexion. When injecting thrombin, care must be taken to avoid occlusion of important vessels and systemic passage of this substance into the circulation [19]. If embolization fails, then open surgery should be considered.

## Conclusion

A pseudoaneurysm in a high-division ATA caused by a retractor following TKA is rare. In the authors’ experience, placement of the retractor along the periosteum and avoidance of deep placement may help to prevent this complication. Once an arterial complication has been diagnosed, it must be treated as soon as possible to prevent potentially devastating complications. Angiography may be a better diagnostic technique because it not only shows the exact location of the injury, but can also be used for therapeutic purposes by stopping the bleeding once discovered. Furthermore, sufficient preoperative assessment and early diagnosis can help to prevent potentially devastating complications.

## Caption Electronic Supplementary Material

Video 1: The pseudoaneurysm was diagnosed by angiography

Video 2: The pseudoaneurysm was treated with high-selectivity microcoil embolization
